# Paramagnetic NMR in drug discovery

**DOI:** 10.1007/s10858-020-00322-0

**Published:** 2020-06-10

**Authors:** Charlotte A. Softley, Mark J. Bostock, Grzegorz M. Popowicz, Michael Sattler

**Affiliations:** 1 Biomolecular NMR and Center for Integrated Protein Science Munich at Department Chemie, Technical University of Munich, Lichtenbergstraße 4, 85747 Garching, Germany; 2 Institute of Structural Biology, Helmholtz Zentrum München, Ingolstädter Landstraße 1, 85764 Neuherberg, Germany

**Keywords:** Nuclear magnetic resonance, Paramagnetism, Pseudo-contact shift, Paramagnetic relaxation enhancement, Drug discovery, Fragment screening, Protein–ligand structure determination

## Abstract

The presence of an unpaired electron in paramagnetic molecules generates significant effects in NMR spectra, which can be exploited to provide restraints complementary to those used in standard structure-calculation protocols. NMR already occupies a central position in drug discovery for its use in fragment screening, structural biology and validation of ligand–target interactions. Paramagnetic restraints provide unique opportunities, for example, for more sensitive screening to identify weaker-binding fragments. A key application of paramagnetic NMR in drug discovery, however, is to provide new structural restraints in cases where crystallography proves intractable. This is particularly important at early stages in drug-discovery programs where crystal structures of weakly-binding fragments are difficult to obtain and crystallization artefacts are probable, but structural information about ligand poses is crucial to guide medicinal chemistry. Numerous applications show the value of paramagnetic restraints to filter computational docking poses and to generate interaction models. Paramagnetic relaxation enhancements (PREs) generate a distance-dependent effect, while pseudo-contact shift (PCS) restraints provide both distance and angular information. Here, we review strategies for introducing paramagnetic centers and discuss examples that illustrate the utility of paramagnetic restraints in drug discovery. Combined with standard approaches, such as chemical shift perturbation and NOE-derived distance information, paramagnetic NMR promises a valuable source of information for many challenging drug-discovery programs.

## Introduction

NMR spectroscopy is well established as a core technique in drug discovery for ligand and fragment screening, validation of target interactions and, in cases where it is not possible to obtain crystal structures of protein–ligand complexes, for structure determination (Hajduk et al. 1999; Gossert and Jahnke 2016; Erlanson et al. 2016). Techniques such as transfer-NOESY (Balaram et al. 1972; Ni 1994), INPHARMA (Sanchez-Pedregal et al. 2005), intermolecular ligand-methyl NOEs (Proudfoot et al. 2017), NMR^2^ (Orts et al. 2016) along with screening techniques such as saturation transfer difference (STD) (Mayer and Meyer 1999), WaterLOGSY (Dalvit et al. 2001), relaxation-editing (Hajduk et al. 1997) and 2D correlation experiments (HSQC, HMQC) (Shuker et al. 1996) are efficient at detecting ligand binding and in some cases the mode of interaction. Importantly, NMR can detect changes of conformation and dynamics upon ligand binding, providing unique opportunities to target allosteric binding sites. A recent impressive example is the use of an NMR-detected conformational assay that enabled the development of novel inhibitors targeting the Bcr-Abl kinase that are currently in advanced clinical trials (Wylie et al. 2017). Many detailed reviews have been published on the role of NMR in structure-based drug discovery (Hajduk et al. 1999; Meyer and Peters 2003; Gossert and Jahnke 2016; Ma et al. 2016; Erlanson et al. 2016; Sugiki et al. 2018; Nitsche and Otting 2018).

Paramagnetic effects offer an opportunity to further enhance and broaden the utility of NMR in drug discovery, providing new approaches for screening and sources of restraints for assessing ligand binding poses and structural analysis. Paramagnetism describes the presence of an unpaired electron in a chemical moiety. NMR spectroscopy is uniquely sensitive to the presence of a paramagnetic center since the strength of magnetic interactions between nuclear and electron spins depends on the involved gyromagnetic ratios, which is about 658 times stronger for an unpaired electron compared to a proton nuclear spin. In solution NMR, paramagnetic effects are mostly exploited through three main phenomena: paramagnetic relaxation enhancement (PRE), pseudo-contact shifts (PCS) and residual dipolar couplings (RDC), although additional mechanisms have also been used to study the structure and dynamics of proteins. The theory and use of paramagnetic NMR has been reviewed elsewhere (Bertini et al. 2002a, 2016; Clore and Iwahara 2009; Otting 2010; Pell et al. 2019; Parigi et al. 2019). Here, we discuss the utility of paramagnetic NMR in solution to overcome challenges in drug discovery, review strategies for introducing paramagnetic centers, and provide perspectives for the future.

**Fig. 1:**
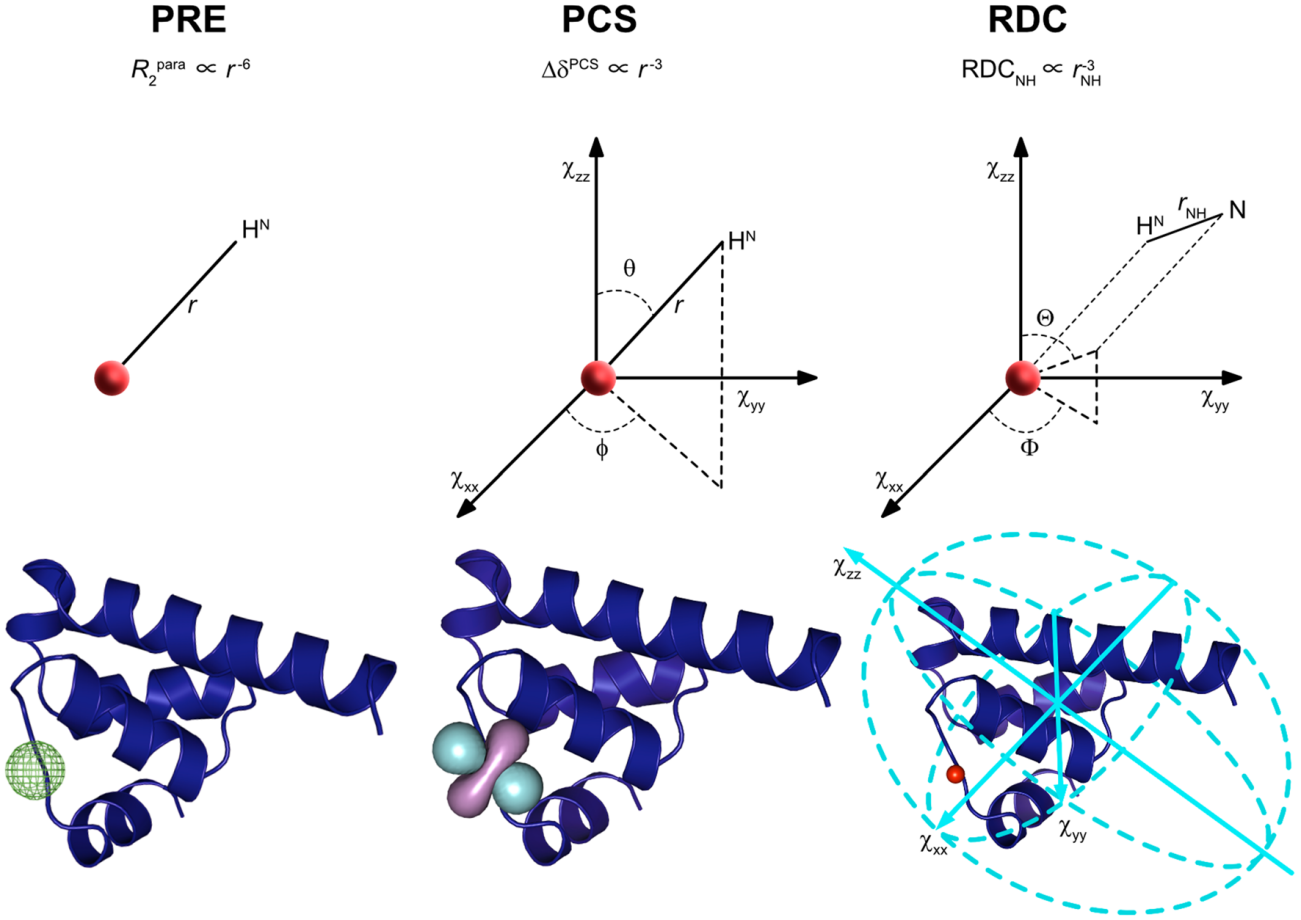
Information available from different types of paramagnetic effects. Paramagnetic relaxation enhancement (PRE) is proportional to the inverse sixth power of the distance between the paramagnetic center and the nucleus of interest (Eqs. 1, 3). Pseudo-contact shifts (PCS) provide information on the distance and the angle of the vector between the atom and the metal ion with respect to the principal axis frame of the magnetic susceptibility anisotropy (Δχ) tensor, which is represented as an isosurface (Eq. 7). Residual dipolar couplings (RDC) provide information on the orientation of a vector connecting two dipolar coupled spins (i.e. the amide bond connecting ^1^H and ^15^N nuclear spins) to the principal axis frame of the metal’s Δχ tensor, represented in light blue on the protein structure (Eq. 8). The paramagnetic center is shown as a red sphere. Isosurfaces were calculated using Paramagpy (Orton et al. 2020)

*Paramagnetic nuclear relaxation* resulting from dipole–dipole interactions between nuclear and electron spins is calculated as the difference in rates between paramagnetic and diamagnetic systems. Two important mechanisms to consider for biomolecular applications are Solomon and Curie relaxation, which depend on the distance *r* between the electron and nuclear spin. Molecular tumbling and associated spectral densities give rise to PRE effects, which are dependent on the distance $\left( r \right)$ between the electronic spin and the nucleus of interest to the inverse sixth power ($r^{ - 6}$) but not orientation (Fig. 1).

The PRE effect due to *the Solomon mechanism* with the spectral density functions written out in full, is given by (Solomon 1955; Solomon and Bloembergen 1956; Clore and Iwahara 2009):

1 $$R_{{2, {\text{SB}}}}^{{{\text{para}}}} = \frac{1}{15}\left( {\frac{{\mu_{0} }}{4\pi }} \right)^{2} \frac{{\gamma_{{\text{I}}}^{2} g^{2} \mu_{{\text{B}}}^{2} S\left( {S + 1} \right)}}{{r^{6} }}\left( {4\tau_{{\text{c}}} + \frac{{3\tau_{{\text{c}}} }}{{1 + \omega_{I}^{2} \left( {\tau_{{\text{c}}} } \right)^{2} }}} \right)$$

where $\mu_{0}$ is the magnetic permeability of free space, $\mu_{{\text{B}}}$ is the Bohr magneton, $\gamma_{{\text{I}}}$ is the gyromagnetic ratio of spin $I$, $\omega_{{\text{I}}}$ is the Larmor frequency of spin $I$, $g$ is the electron $g$-factor, $S$ is the spin quantum number and $\tau_{{\text{c}}}$ is the correlation time given by:

2 $$\frac{1}{{\tau_{{\text{c}}} }} = \frac{1}{{\tau_{{\text{r}}} }} + \frac{1}{{\tau_{{\text{s}}} }} + \frac{1}{{\tau_{{\text{m}}} }}$$

$\tau_{{\text{r}}}$ is the rotational correlation time of the paramagnetic protein or complex; $\tau_{{\text{s}}}$ is the lifetime (effective relaxation time) of the electron spin and $\tau_{{\text{m}}}$ is the lifetime of the complex. $\tau_{{\text{m}}}$ is typically long relative to the other terms so depending on the source of paramagnetism, $\tau_{{\text{c}}}$ may be dominated by $\tau_{{\text{r}}}$ (long $\tau_{{\text{s}}}$ e.g. nitroxide radicals) or by $\tau_{{\text{s}}}$ (where $\tau_{{\text{s}}} \ll \tau_{{\text{r}}}$), for example some paramagnetic transition metals and most lanthanides (Jahnke 2002; Clore and Iwahara 2009).

In addition to paramagnetic relaxation due to the Solomon mechanism ($R_{{2,{\text{SB}}}}^{{{\text{para}}}}$), the presence of an external magnetic field leads to differing populations of the $S$ and $I$ spin energy levels according to the Boltzmann distribution (splitting is given by $M_{{\text{S}}}$ and $M_{{\text{I}}}$ respectively) (Bertini et al. 2002a, b). Dipole–dipole interaction between the nuclear spins and the thermal average of the total electronic magnetic moment $\langle S_{z} \rangle$, leads to a further relaxation contribution, *Curie spin relaxation* or magnetic susceptibility relaxation. For transverse relaxation, this is given as (Gueron 1975; Vega and Fiat 1976; Bertini et al. 2002a):

3 $$R_{{2, {\text{Curie}}}}^{{{\text{para}}}} = \frac{1}{5}\left( {\frac{{\mu_{0} }}{4\pi }} \right)^{2} \frac{{\gamma_{I}^{2} g^{2} \mu_{B}^{2} \langle S_{z} \rangle ^{2} }}{{r^{6} }}\left( {4\tau_{c}^{{{\text{Curie}}}} + \frac{{3\tau_{{\text{c}}}^{{{\text{Curie}}}} }}{{1 + \omega_{I}^{2} \left( {\tau_{{\text{c}}}^{{{\text{Curie}}}} } \right)^{2} }}} \right)$$

where $\langle S_{z} \rangle$ is the expectation value of $S_{z}$. Note, that Eq. 3 assumes an isotropic magnetic susceptibility tensor. Corrections need to be applied for anisotropic magnetic susceptibility (Bertini et al. 2002a). Assuming isotropic molecular rotation, $\langle S_{z} \rangle$ can be expanded to first order as (Bertini et al. 2002a; Parigi et al. 2019):

4 $$\langle S_{z} \rangle = - \frac{{g\mu_{B} S\left( {S + 1} \right)B_{0} }}{3kT}$$

and the Curie law may be used to rewrite Eqs. 3 and 4 as (Bertini et al. 2002a; Walder et al. 2018; Parigi et al. 2019):

5 $$R_{{2, {\text{Curie}}}}^{{{\text{para}}}} = \frac{1}{5}\left( {\frac{1}{4\pi }} \right)^{2} \frac{{\omega_{I}^{2} \chi_{{{\text{iso}}}}^{2} }}{{r^{6} }}\left( {4\tau_{{\text{c}}}^{{{\text{Curie}}}} + \frac{{3\tau_{{\text{c}}}^{{{\text{Curie}}}} }}{{1 + \omega_{I}^{2} \left( {\tau_{{\text{c}}}^{{{\text{Curie}}}} } \right)^{2} }}} \right)$$

Since the interaction is with the ensemble averaged electron spin, $\langle S_{z} \rangle^{2}$, the electronic correlation time is no longer considered and the correlation time ($\tau_{{\text{c}}}^{{{\text{Curie}}}} )$ is given as:

6 $$\frac{1}{{\tau_{{\text{c}}}^{{{\text{Curie}}}} }} = \frac{1}{{\tau_{{\text{r}}} }} + \frac{1}{{\tau_{{\text{m}}} }}$$

**Fig. 2:**
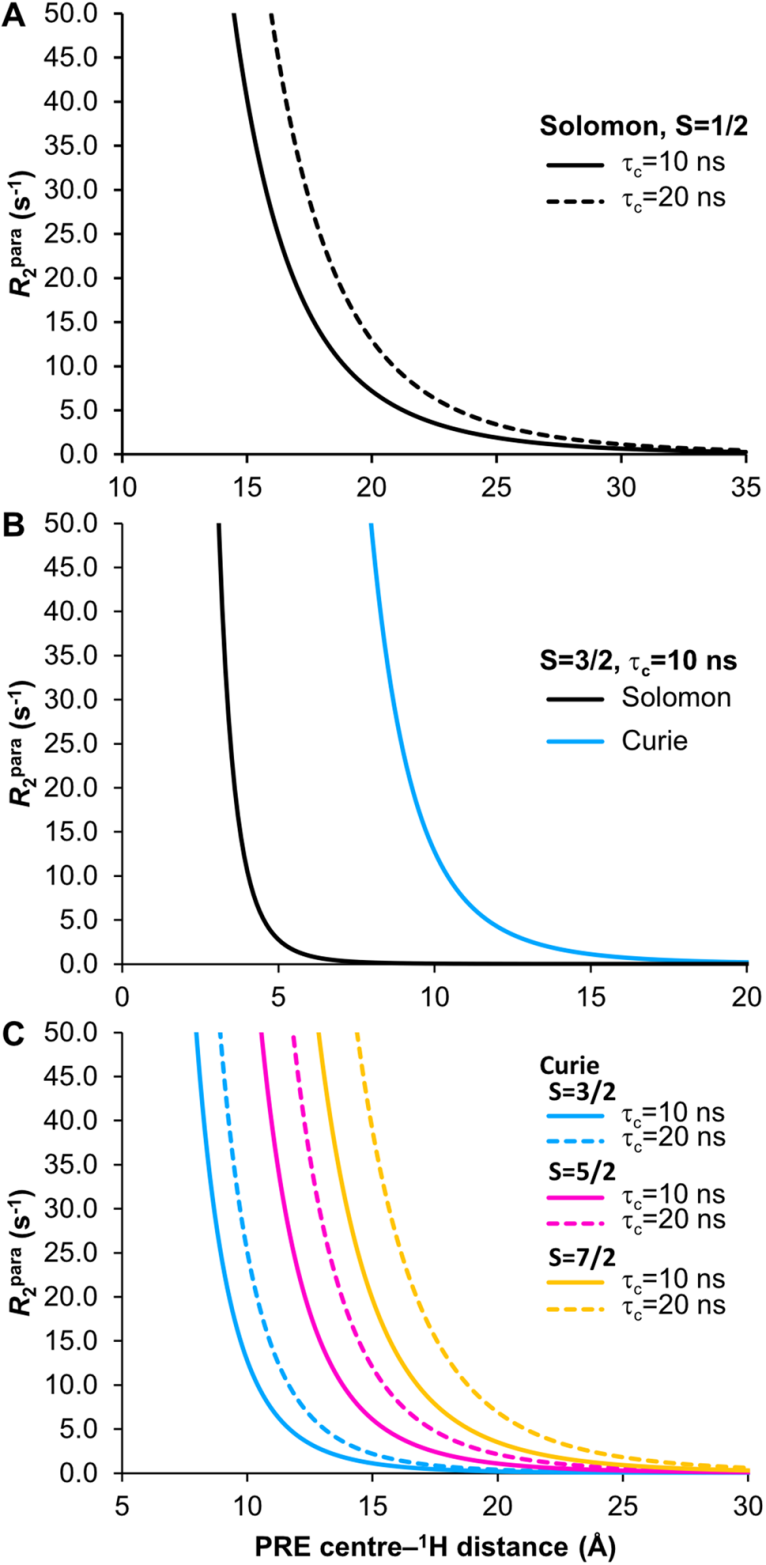
Relaxation effects in paramagnetic systems due to Solomon and Curie mechanisms. **a** For systems with a slow electronic relaxation time ($$\tau_{{\text{s}}}$$) e.g. nitroxide radicals $$\tau_{{\text{s}}} \sim 100$$ ns, transverse paramagnetic relaxation is dominated by the Solomon mechanism (Eq. 1). Relaxation enhancements relative to $$R_{2}^{{{\text{dia}}}}$$ are seen typically between 10–20 Å (Table 1). Curves are shown for a spin $$\raise.5ex\hbox{$\scriptstyle 1$}\kern-.1em/ \kern-.15em\lower.25ex\hbox{$\scriptstyle 2$}$$ particle. Tumbling is dominated by $$\tau_{{\text{c}}}$$ (10 or 20 ns) and the complex lifetime $$\tau_{{\text{m}}}$$ is assumed to be long relative to $$\tau_{{\text{c}}}$$. **b** For particles with a faster $$\tau_{{\text{s}}}$$ e.g. many lanthanide metals, the Curie relaxation mechanism dominates over the Solomon contribution, which is insignificant in the range of interest. Curves were simulated using Eqs. 1, 3 and 4, using $$\tau_{{\text{s}}} = 10^{ - 13}$$ s and $$\tau_{{\text{c}}} = 10$$ ns. S = 3/2 for example for cobalt (II). **c** Curie relaxation is illustrated for different spins states (S = 3/2, 5/2, 7/2) and different correlation times $$\tau_{{\text{c}}}$$ = 10 or 20 ns. Other values are the same as for the Curie contribution in (**b**). The Curie contribution is significant between around 3 and 10 Å (see Table 1). Simulations are for ^1^H relaxation at 600 MHz (^1^H frequency) and 298 K

Solomon relaxation typically dominates for nitroxide radicals and metal ions with isotropic or near-isotropic magnetic susceptibility, which have slower relaxing electronic spins, e.g. Mn^2+^, Gd^3+^ (Fig. 2a). Contributions from Curie relaxation are typically negligible as $\langle S_{z} \rangle ^{2}$ is much smaller than $S\left( {S + 1} \right)/3$ in Eq. 1. However, the Curie contribution scales with $\tau_{{\text{r}}}$ and magnetic field strength, making this effect more significant for large proteins and at higher magnetic fields. This dominates over the Solomon relaxation when $\tau_{{\text{s}}}$ is much faster than $\tau_{{\text{r}}}$ (Fig. 2b). Curie relaxation is therefore dominant for metal ions with fast relaxing electronic spins e.g. Yb^3+^, Dy^3+^ (Pintacuda et al. 2004; Bertini et al. 2016) with a dependence on the spin quantum number (Fig. 2c). Relaxation contributions from the Curie and Solomon mechanisms are illustrated in Fig. 2 and relaxation enhancements relative to diamagnetic transverse relaxation are shown in Table 1. Relative to $R_{2}^{{{\text{dia}}}}$, enhancements due to the Solomon mechanism, assuming a spin ½ nucleus, are observed between 10 and 20 Å. Above this range, no significant enhancement is observed and below 10 Å, signal bleaching occurs. For relaxation dominated by the Curie mechanism, effects are seen below ca. 10 Å with substantial effects leading to bleaching below ca. 5 Å, depending on the spin quantum number.

**Table 1:** $R_{2}$ enhancement factors, following Bertini et al. (2004), are calculated for Solomon (a) and Curie (b) contributions to paramagnetic relaxation for different correlation times ($\tau_{{\text{c}}}$) and spin states ($S$) as a function of the distance between the paramagnetic center (an unpaired electron e.g. in a nitroxide radical (a) or a metal centre (b)) and a proton spin. Calculations are for 600 MHz (^1^H frequency) and 298 K, using Eqs. 1, 3 and 4 as described in the legend to Fig. 2 and assume $R_{2}^{{{\text{dia}}}}$ values of 60 s^−1^ and 120 s^−1^ for $\tau_{{\text{c}}}$ of 10 ns and 20 ns respectively

(a)	Solomon	
	$$\left( {R_{2}^{{{\text{para}}}} + R_{2}^{{{\text{dia}}}} } \right)/R_{2}^{{{\text{dia}}}}$$	
Electron-^1^H (Å)	$\tau_{{\text{c}}} = 10$ ns	$\tau_{{\text{c}}} = 20$ ns
3	10,509.60	9474.37
4	1871.30	1687.06
5	491.29	442.99
6	165.20	149.02
7	66.12	59.70
8	30.22	27.34
9	15.42	14.00
10	8.66	7.91
11	5.32	4.90
12	3.57	3.31
13	2.59	2.43
14	2.02	1.92
15	1.67	1.61
16	1.46	1.41
17	1.32	1.29
18	1.23	1.20
19	1.16	1.15
20	1.12	1.11
30	1.01	1.01

(b)	Curie					
	$$\left( {R_{2}^{{{\text{para}}}} + R_{2}^{{{\text{dia}}}} } \right)/R_{2}^{{{\text{dia}}}}$$					
	$\tau_{{\text{c}}} = 10$ ns			$\tau_{{\text{c}}} = 20$ ns		
Metal-^1^H (Å)	$S = 3/2$	$S = 5/2$	$S = 7/2$	$S = 3/2$	$S = 5/2$	$S = 7/2$
3	292.08	1585.77	5135.65	287.77	1562.32	5059.67
4	52.81	283.05	914.86	52.04	278.88	901.33
5	14.58	74.94	240.56	14.38	73.84	237.02
6	5.55	25.76	81.23	5.48	25.40	80.04
7	2.80	10.82	32.82	2.78	10.67	32.35
8	1.81	5.41	15.28	1.80	5.34	15.07
9	1.40	3.17	8.04	1.39	3.14	7.94
10	1.21	2.16	4.74	1.21	2.14	4.69
20	1.00	1.02	1.06	1.00	1.02	1.06
30	1.00	1.00	1.01	1.00	1.00	1.01

In contrast to the PRE, the *pseudocontact shift (PCS)* effect leads to changes in chemical shift positions. Nuclei sense the sum of the external magnetic field and of a field caused by the electron static magnetic moment. Therefore, the dipolar interaction between the total magnetic field and nuclei is not completely averaged by molecular rotation (in contrast to dipole–dipole interactions between nuclear spins). The anisotropy of the static magnetic moment yields average residual dipolar interactions, which cause the PCS effect. As a result, PCS depends on both the distance ($r^{ - 3}$) and the angle ($\theta ,\phi$) relative to the principle axis frame of the metal’s magnetic susceptibility anisotropy tensor (${\Delta }\chi$), given as axial $\left( {{\Delta }\chi_{{{\text{ax}}}} = \chi_{{\text{z}}} - \left( {\chi_{{\text{x}}} + \chi_{{\text{y}}} } \right)/2} \right)$ and rhombic $\left( {{\Delta }\chi_{{{\text{rh}}}} = \chi_{{\text{x}}} - \chi_{{\text{y}}} } \right)$ components (Fig. 1). This angular dependence makes PCS measurements a particularly rich source of structural information. Several studies have shown their use in structure calculations (Tu and Gochin 1999; Pintacuda et al. 2006; Saio et al. 2010; Schmitz et al. 2012; Yagi et al. 2013; Hass and Ubbink 2014; Crick et al. 2015) and refinements (Banci et al. 1996; Bertini et al. 2009). The PCS is given by (McConnell and Robertson 1958):

7 $${\Delta }\delta^{{{\text{PCS}}}} = \frac{1}{{12\pi r^{3} }}\left[ {{\Delta }\chi_{{{\text{ax}}}} \left( {3\cos^{2} \theta - 1} \right) + 1.5{\Delta }\chi_{{{\text{rh}}}} \sin^{2} \theta \cos 2\phi } \right]$$

**Fig. 3:**
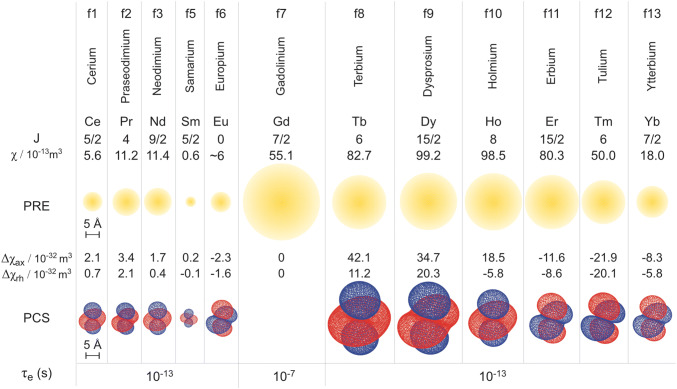
Properties of various paramagnetic, non-radioactive lanthanides. For each metal, the electron angular momentum $\left( J \right)$ is given. The PRE contribution is shown as a yellow isosurface corresponding to paramagnetic broadening of the ^1^H NMR signals by 80 Hz at 800 MHz for a protein with a rotational correlation time of 15 ns. Representative PCS isosurfaces (blue/red positive/negative) are shown for pseudocontact shifts of ± 5 ppm using the Δχ tensors for calbindin D_9k_ (Bertini et al. 2001). Electronic relaxation times $\left( {\tau_{{\text{e}}} } \right)$ are given for 18.8 T (Alsaadi et al. 1980). Reprinted with permission from reference (Pintacuda et al. 2007). Copyright 2007 American Chemical Society

While all paramagnetic centers produce a PRE effect of varying magnitude the PCS is limited to metal ions with a non-zero magnetic susceptibility anisotropy tensor, ${\Delta }\chi$. Lanthanide metal ions are frequently used to induce PCS effects. Careful choice of the lanthanide ion allows tuning of the relative magnitudes of the PRE effect and the PCS effect (Fig. 3), ranging from gadolinium (${\Delta }\chi_{{{\text{ax}}}} = {\Delta }\chi_{{{\text{rh}}}} = 0$), which exhibits only a strong PRE effect and is thus frequently used as a contrast agent in magnetic resonance imaging (MRI), to terbium and dysprosium which show large PCS effects and more modest PRE.

*Residual dipolar coupling (RDC)* arises in the case of anisotropic magnetic susceptibility of the paramagnetic center, which induces partial self-alignment of the molecules relative to the magnetic field. When a molecule tumbles freely and isotropically, dipole–dipole interactions are averaged to zero; in the case of partial alignment, an RDC remains, providing orientation information about scalar-coupled pairs of spins, relative to the alignment tensor. The RDC for two spins, 1 and 2 is defined in Eq. 8, where $\hbar$ is the reduced Planck’s constant, $B_{0}$ the external field strength, $r_{12}$ the internuclear distance between the two spins, $k_{{\text{B}}}$ the Boltzmann constant, and $T$ the temperature; ${\Theta }$ and ${\Phi }$ are as defined relative to the components of the anisotropic magnetic susceptibility as in Fig. 1 (Bertini et al. 2002b; Otting 2010):

8 $${\text{RDC}}_{12} \left( {{\text{Hz}}} \right) = - \frac{1}{4\pi }\frac{{B_{0}^{2} }}{{15k_{{\text{B}}} T}}\frac{{\hbar \gamma_{1} \gamma_{2} }}{{2\pi r_{12}^{3} }} \times \left[ {\Delta \chi_{{{\text{ax}}}} \left( {3{\cos}^{2} \Theta - 1} \right) + 1.5\Delta \chi_{{{\text{rh}}}} {\sin}^{2} \Theta {\cos}2\Phi } \right]$$

Residual dipolar couplings have been extensively reviewed elsewhere (Bax et al. 2001; Prestegard et al. 2004; Blackledge 2005; Chen and Tjandra 2012).

All of these observables (and additional effects, for example, from cross-correlation between dipolar and Curie interactions (Bertini et al. 2002b)) have been exploited in biomolecular structure determination (Battiste and Wagner 2000; Hus et al. 2000; Prestegard et al. 2004; Bertoncini et al. 2005; Volkov et al. 2006; Simon et al. 2010; Saio et al. 2010; Hennig and Sattler 2014; Crick et al. 2015; Sjodt and Clubb 2017). However, it is mainly PRE and PCS measurements that are used in early stage drug discovery, and on which this review will focus. RDCs are used primarily to validate PCS restraints or in the calculation and validation of the anisotropy tensor.

## Paramagnetic tags for protein conjugation

In order to exploit paramagnetic effects, a paramagnetic center is required. With the exception of proteins that harbor an intrinsic metal binding site, which can bind a paramagnetic ion (Burroughs et al. 1994; Pidcock and Moore 2001; Bertini et al. 2002b), most proteins require the artificial addition of a paramagnetic center via a tag. The variety of tags is large and has recently been thoroughly reviewed (Joss and Häussinger 2019). A key criterion for all tags is limited mobility; otherwise motional averaging reduces the magnitude of the paramagnetic effects and leads to inaccurate or motional-averaging of distance and orientation-dependent effects. Here, we discuss a selection of commonly used tags and some of those employed in the examples covered in this review.

**Fig. 4:**
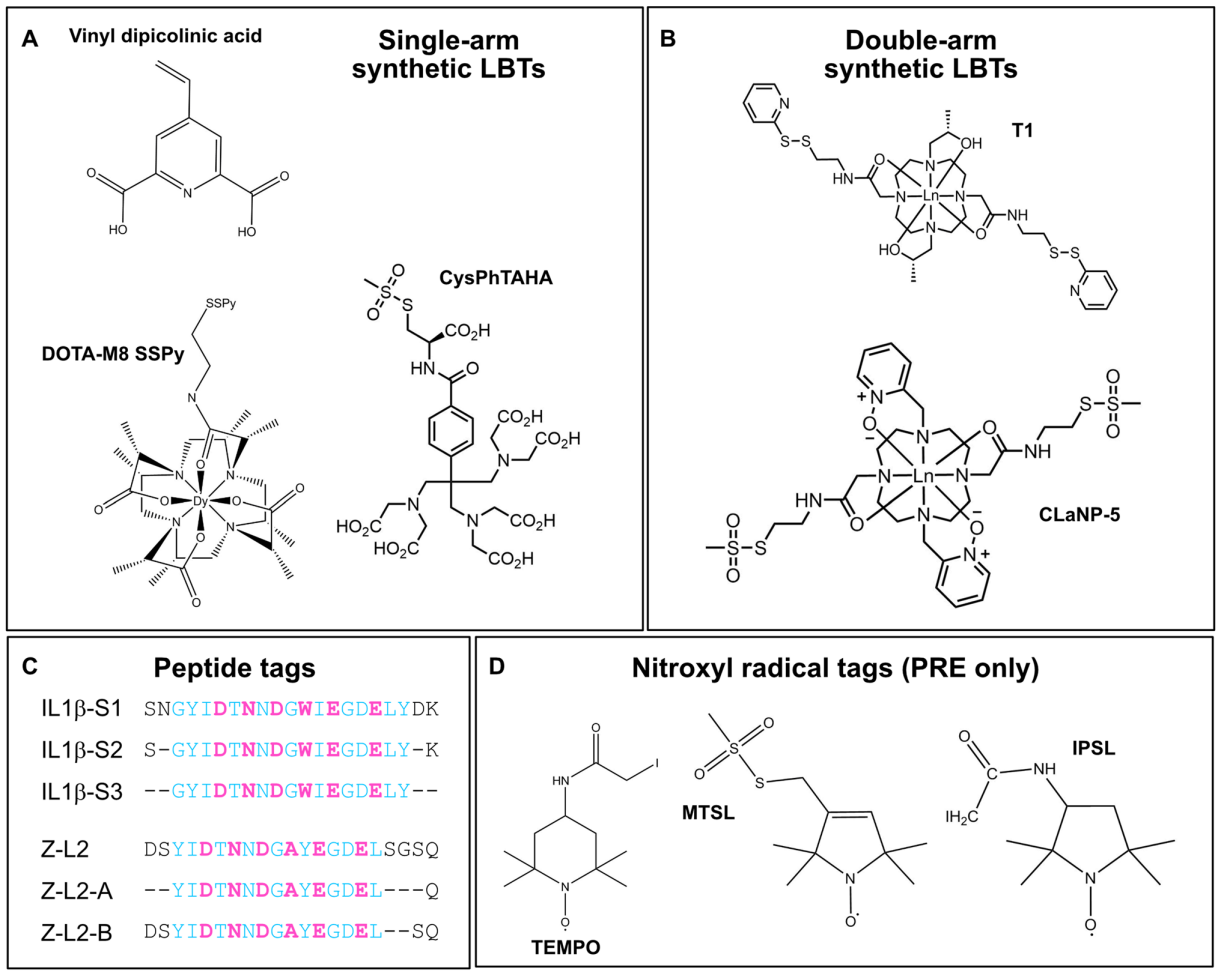
Structures of selected paramagnetic tags discussed in this work. **a** Single-arm synthetic lanthanide binding tags (LBTs). **b** Double-arm synthetic LBTs. **c** Lanthanide binding peptide sequences used in Barthelmes et al. (2011) and Barb and Subedi (2016). Residues marked in magenta interact with the lanthanide ion. Residues in cyan form part of the lanthanide binding peptide sequence. Residues in black are flanking residues from the native protein loop sequence, with the number of flanking residues varied to produce different tags. **d** Nitroxyl radical tags. **a**–**c** Can be used to introduce PCS, PRE and RDC effects, depending on the metal used; **d** are used solely for PRE

The earliest examples of the conjugation of spin labels involved nitroxide spin labels, among the smallest tags, introduced by the Hubbell lab for use in EPR (Todd et al. 1989) (Fig. 4). These consist of a nitroxide group, attached to bulky quaternary carbons to prevent quenching of the radical (Roser et al. 2016). They are normally conjugated to a cysteine by a disulfide bridge (Ajtai et al. 1990; Battiste and Wagner 2000) and are used for PREs due to their isotropic susceptibility tensor. IPSL (N-(1-oxyl-2,2,5,5-tetramethyl-3-pyrrolidinyl)iodoacetamide) conjugation with cysteines produces thioether bonds, which are chemically more stable than the disulfide bonds formed by MTSL (1-oxyl-2,2,5,5-tetramethylpyrroline-3-methyl)methanethiosulfonate) and thus preferred (Göbl et al. 2014). Alternatives include other PROXYL-based (2,2,5,5-tetramethyl-3-pyrrolidine-N-oxyl) tags (Gillespie and Shortle 1997; Pavićević et al. 2017), which can be adapted to bind to lysine residues, as well as lipids and nucleic acids (Keana et al. 1976; Barnwal et al. 2017). One disadvantage of these molecules is that their flexibility and the types of motion they undergo are strongly dependent on their local environment (Lietzow and Hubbell 2004; López et al. 2012). While this can be useful for studying the environment of the tags, a preferably rigid and well-defined tag position is required for generation of structural restraints. Rotamer libraries of spin labels attached to amino acid side chains have been modeled for analysis and in silico calculations (Kroncke et al. 2010; Polyhach et al. 2011; Freed et al. 2011).

For PCS studies, an anisotropic magnetic susceptibility tensor is required, which can be provided by paramagnetic metal ions, such as ytterbium and thulium (Fig. 3). These can be attached using lanthanide binding tags (LBTs), e.g. (i) chemically-synthesized metal chelating tags that are conjugated to solvent-accessible cysteine(s); (ii) peptide tags that coordinate the metal, or (iii) via direct binding of the metal to histidine residues. LBTs have been thoroughly reviewed recently (Nitsche and Otting 2017; Joss and Häussinger 2019). In the following, we provide a brief summary.

Single-armed cysteine-linked LBTs include dipicolinic acid (Su et al. 2006, 2008) and cysteinyl-phenyl-triaminohexaacetate (Cys-Ph-TAHA) (Peters et al. 2011), as well as DOTA-type tags, including DOTA-M8 (Häussinger et al. 2009) and DOTA-M7FPy (Müntener et al. 2018) (Fig. 4). DOTA-style LBTs are synthesized pre-chelated with metal ions, while TAHA and dipicolinic acid are easily accessible by chemical synthesis on the scale of hundreds of milligrams (Su et al. 2008; Peters et al. 2011) and the metal is added after protein conjugation, allowing chelation of a range of paramagnetic metals. Of importance is the rigidity of the tag, which corresponds to a larger effective magnetic anisotropy tensor and, by extension, greater paramagnetic effects—in this case, out of the single-arm tags, the DOTA-type tags have been shown to be more rigid and to give rise to larger tensors (Joss and Häussinger 2019). Earlier versions of the DOTA tags suffered from exchange between two coordinating geometries (square antiprism and twisted-square antiprism), but this problem was reduced by further functionalization (Polášek et al. 2004; Häussinger et al. 2009; Liu et al. 2014). Some LBTs have free coordination sites for either water or other groups, such as carboxylic acid side chains (Su et al. 2008; Swarbrick et al. 2011; Peters et al. 2011; Lee et al. 2015). This can lead to additional dynamics in the system and can impact the effective tensor obtained. However, this can also be of benefit since, with the availability of a residue such as aspartic acid on the surface of the protein nearby, this can stabilize and rigidify the position of the paramagnetic center and thereby increase the observed paramagnetic effects (Su et al. 2008; Lee et al. 2015).

**Fig. 5:**
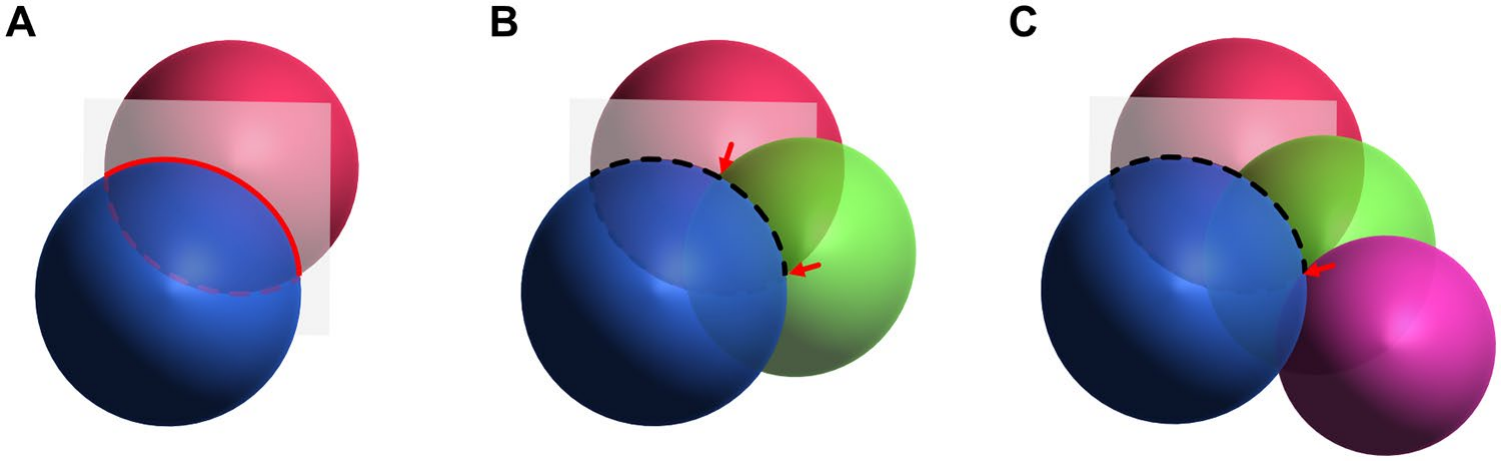
Localization of a ligand via PCS restraints requires multiple tensor isosurfaces located at different positions, illustrated by overlapping spheres. The intersection between two spheres is a circle (**a**). Introducing a third isosurface reduces this to two points of intersection (red arrows) (**b**). Unambiguous localization requires four isosurfaces (red arrow) (**c**). In reality, more positions may be required, for example as a result of tag motion. It may also be possible to exclude some positions based on chemical and structural considerations

An elegant method to rigidify the position and orientation of the metal relative to the protein is to use a two-armed tag, binding at two cysteines, e.g. CLaNP-5 (Keizers et al. 2007) and the T1 and T2 tags from Lee et al. (Lee et al. 2016), leading to large susceptibility anisotropy and alignment tensors (Fig. 4). CLaNP-7 has been developed with a smaller total charge than CLaNP-5 in order to reduce any effect on the electrostatic potential of the protein (Liu et al. 2012). This tag, however, is pH-dependent in the presence of a histidine residue in its vicinity, hypothetically due to the imidazole interacting with a water (or hydroxide ion) bound to the vacant coordination site. This leads to multiple NMR signals at some pH values. Two-armed tags that bind transition metals are also available (Miao et al. 2019). As transition metals have smaller anisotropy tensors, these are more applicable to systems where the tagging site is close to the binding site. Due to the fourfold degeneracy of the anisotropy tensors, as well as the flexibility of the tag linker and protein and any errors in the measurements, multiple sites for LBT attachment are usually required to unambiguously localize, for example, a ligand binding site (Bertini et al. 2016) (Fig. 5). For small ligands, this is particularly challenging as, even for a fully asymmetric tensor, multiple positions in space can have the same PCS magnitude, leading to degenerate positions. For larger ligands, this is less of a problem as there are more atoms involved, leading to a wider variation in shifts across the molecule and fewer sites on the protein that would match the necessary gradients. Even for small ligands though, the use of multiple tensors or tag positions can be used to triangulate the position of the ligand and additionally, chemical and structural considerations can be used to remove impossible or unlikely solutions (John et al. 2006). The different orientations of the tensors from CLaNP and T1/T2 tags could lead to complementary data in this way, while using only one set of mutations for the binding of the tag. The same is true of the T1 and T2 tag pair, which are enantiomers and therefore provide different tensors. In the case that these tensors have a high intersection angle (close to orthogonal) to one another, the use of both tags at the same site can reduce this degeneracy. A potential disadvantage of double cysteine tags is the requirement for two cysteines within a suitable distance for tagging, which typically must be introduced by mutagenesis. This can be challenging depending on the protein studied and may impact on protein stability and function.

In some cases, cysteines are key to the function or fold of biomolecules and are therefore impossible to mutate out or tag. Therefore, strategies have been developed to avoid the reliance on cysteine residues. These include peptide tags and histidine chelation. Double histidine chelation requires two histidine residues, situated at $i$ and $i + 4$ on an α-helix or $i$ and $i + 2$ on a β-strand, which directly chelate the metal. Again, mutations are often required for this, but the technique has been shown to work with copper ions, using iminodiacetic acid as a ‘lid’ to fill outstanding chelation vacancies on the metal (Cunningham et al. 2015) or simply with cobalt ions chelating the histidines without additional molecules for chelation (Bahramzadeh et al. 2018). For this technique, in order to chelate only the desired metal, any existing metal is first removed with EDTA, which the protein must be able to withstand.

Metal-binding peptides may be introduced to enable genetic encoding of the metal-binding site for recombinant protein production. Peptide sequences, based on calcium-binding motifs, have been iteratively evolved to bind selectively and with high affinity (*K*_d_ ≈ nM) to lanthanide metals (Nitz et al. 2003). Early tags were attached to the N- and C-termini of proteins, with rigidification in some cases provided by cross-linking to a cysteine (Saio et al. 2009). However, this still requires further protein modifications and the availability of an appropriate cysteine. Other strategies involve a two-point anchored peptide LBT, via insertion in a protein loop. Although this risks disrupting the protein fold, several studies have shown that with careful choice of tag and insertion location, this can be achieved with minimal structural change. A detailed study of IL1*β* incorporated a 17-residue LBT sequence in three different loops with variable flanking residues and mutation of existing loop residues (Fig. 4) (Silvaggi et al. 2007; Barthelmes et al. 2011). The observation of larger RDCs compared to samples with single-attachment peptide LBTs and good agreement between predicted and back-calculated PCSs indicate its rigidity. A crystal structure shows that the IL1*β* fold is preserved although slightly higher dynamics are observed in the LBT region from Lipari-Szabo analysis (Lipari and Szabo 1982a, b) of ^15^N NMR relaxation data, not unexpected for a loop region. An interesting advantage of such tags for X-ray crystallography is that phasing information can be determined from the presence of the heavy metal ion (Silvaggi et al. 2007). This is useful where similar structures are not available for molecular replacement, or when molecular replacement alone gives poor results (Panjikar et al. 2009). Similar results have been shown for immunoglobulin G (IgG) binding proteins with insertion into a loop between two helices (Fig. 4) (Barb et al. 2012; Barb and Subedi 2016). Compared to the loop used by Barthelmes et al. (Barthelmes et al. 2011) the C-terminal residues are removed and the Trp is replaced by Ala, as the observation of two indole NH signals indicated multiple side-chain conformations for the tryptophan (Su et al. 2006; Barb et al. 2012). RDCs were used to confirm correlation with existing structures and a structure of the protein with bound lanthanide ions was determined using Xplor-NIH (Banci et al. 2004; Schwieters et al. 2006). As expected, motion of the LBT domain was observed relative to the core of the protein and therefore improvements were made to the LBT by removal of flanking residues leading to a further reduction in tag mobility, and a consequent increase in tensor magnitude (Barb and Subedi 2016).

It is also possible to introduce paramagnetic centers via unnatural amino acids, which may already incorporate a paramagnetic center (Schmidt et al. 2014) or bind specifically to a paramagnetic tag (Fleissner et al. 2009; Loh et al. 2015; Kugele et al. 2019). These have the disadvantage of requiring the introduction of an unnatural amino acid, usually via Amber codon suppression, which typically involves some optimization of expression protocols. However, a significant advantage is that native cysteines, which may be required for functional activity, can be preserved. This approach is currently not commonly used in drug discovery but may present a potential area for future development. The reader is referred to relevant papers for further information (Cellitti et al. 2008; Jones et al. 2010; Liu et al. 2014; Lang et al. 2015; Braun et al. 2019).

Having successfully introduced a paramagnetic center, as discussed above, multiple applications are possible to aid the drug discovery process, which are discussed further below and summarized in Table 2.

**Table 2:** Examples of paramagnetic restraints used in detecting ligand–protein interactions and drug discovery applications

Protein	PCS or PRE?	Tag	Metal	Calculation method^#^	Application	References
Bcl-xL	PRE	TEMPO-adduct	n.a	n.a	Second-site screening	Jahnke et al. (2000)
FK506 binding protein (FKBP)	PRE	Spin-labeled lysines	n.a	n.a	Primary NMR screening	Jahnke et al. (2001)
HIV-1 gp41	PRE & PCS	MTSL- or EDTA-labeled C-peptide	Co^2+^	DOCK & Xplor-NIH	Second-site screening and paramagnetic assisted selection of docking poses	Balogh et al. (2009)
HIV-1 gp41	PRE	MTSL-labeled C-peptide	n.a	AutoDock 4.2, AutoDock Vina & Xplor-NIH	Paramagnetic assisted selection of docking poses	Gochin et al. (2011)
*E. coli* DNA polymerase III ε-exonuclease NTD & θ-subunit	PCS	Intrinsic metal center	Dy^3+^/Tb^3+^/Er^3+^	Xplor-NIH	PCS-calculated binding-mode of thymidine (fast exchange)	John et al. (2006)
Grb2-SH2	PRE & PCS	N-terminal anchored LBT, linked to M73C mutation	Dy^3+^/Tb^3+^/Tm^3+^	Xplor-NIH	PRE-based screening (using Gd^3+^); PCS-calculated binding mode of pYTN peptide (low affinity, fast exchange) & macrocyclic inhibitor (high affinity, slow exchange)	Saio et al. (2011)
BRM bromodomain	^19^F PCS	4MMpyMTA linked to K64C or L86C	Tb^3+^/T^3+^	DOCK6 UCSF, HADDOCK	^19^F-PCS-CEST for ligand in intermediate exchange Filtering of docked conformations using ^19^F-PCS or HADDOCK docking with PCS restraints	Gao et al. (2017)
DENPro	PCS	C2-lanthanide tag linked to A57C, S34C or S68C	Tm^3+^/Tb^3+^	AutoDock Vina & Rosetta	*Tert-*butyl derived ligand (slow exchange). NOESY cross-peaks to intense *tert*-butyl & NOEs to other ligand protons for ${\Delta }\delta^{{{\text{PCS}}}}$	Chen et al. (2016)
FKBP12	PCS	CLaNP-5	Yb^3+^	Xplor-NIH with PARArestraints	PCS-calculated binding mode of fragments (fast exchange). Knowledge of *K*_d_ to estimate δ_bound_	Guan et al. (2013)
BRM bromodomain	PCS	4MMpyMTA linked to K64C	Tm^3+^	AutoDock 4.2	Relaxation dispersion to measure bound ^1^H-chemical shifts in intermediate exchange. PCS-filtering of docked conformations	Xu et al. (2018b)
Human carbonic anhydrase	^19^F-PCS	DOTA-M8-SSPy and Ln-M7PyThiazole-DOTA	Tm^3+^	Least-squares calculation of ^19^F position	^19^F-labeled sulphonamide inhibitors (slow exchange)	Zimmermann et al. (2019)

^#^References to the software mentioned in this table can be found in Table 3

## PRE in drug discovery

As shown in Eqs. 1 and 3, the PRE for a given nuclear spin depends on the distance to the paramagnetic center ($r^{ - 6}$) but not the angle (Fig. 1). As a result, the structural information provided has a shorter range and is not as rich as PCS data, which is distance and orientation dependent and decays with $r^{ - 3}$; however, the PRE effect is still readily exploited in drug discovery.

PREs can be used to enhance the sensitivity of drug screening, either by introducing a paramagnetic center on the protein or via a ligand known to bind the target protein of interest. The paramagnetic center induces a PRE effect on any interacting molecules introducing a relaxation effect that depends on the distance, $r$, as well as the exchange rate $k_{{{\text{ex}}}}$ of the complex and the residence time $\tau_{{\text{m}}} = k_{{{\text{off}}}}^{ - 1}$ of the ligand, although the latter is typically ignored as discussed earlier (Eqs. 2, 4) (Clore and Iwahara 2009). Exchange effects can be accounted for by considering the McConnell equations (McConnell 1958; Clore and Iwahara 2009). It has previously been demonstrated that a weakly populated minor state (with a short distance *r*, and thus a very strong PRE) can be identified by intermolecular PREs, as long as the exchange rate is larger than the PRE enhancement and the chemical shift difference between two states, i.e. in fast exchange on the chemical shift and relaxation timescales (Iwahara and Clore 2006; Clore and Iwahara 2009). In the case of small molecules e.g. ligands, the considerably faster $\tau_{{\text{c}}}$ of the free ligand means that exchange contributions do not make a significant additional contribution to the transverse relaxation rate. Thus for ligand-observed experiments, the PRE effect experienced by the ligand is scaled by the population of the protein–ligand complex as discussed below (Eq. 9) (Jahnke 2002; John et al. 2006).

**Fig. 6:**
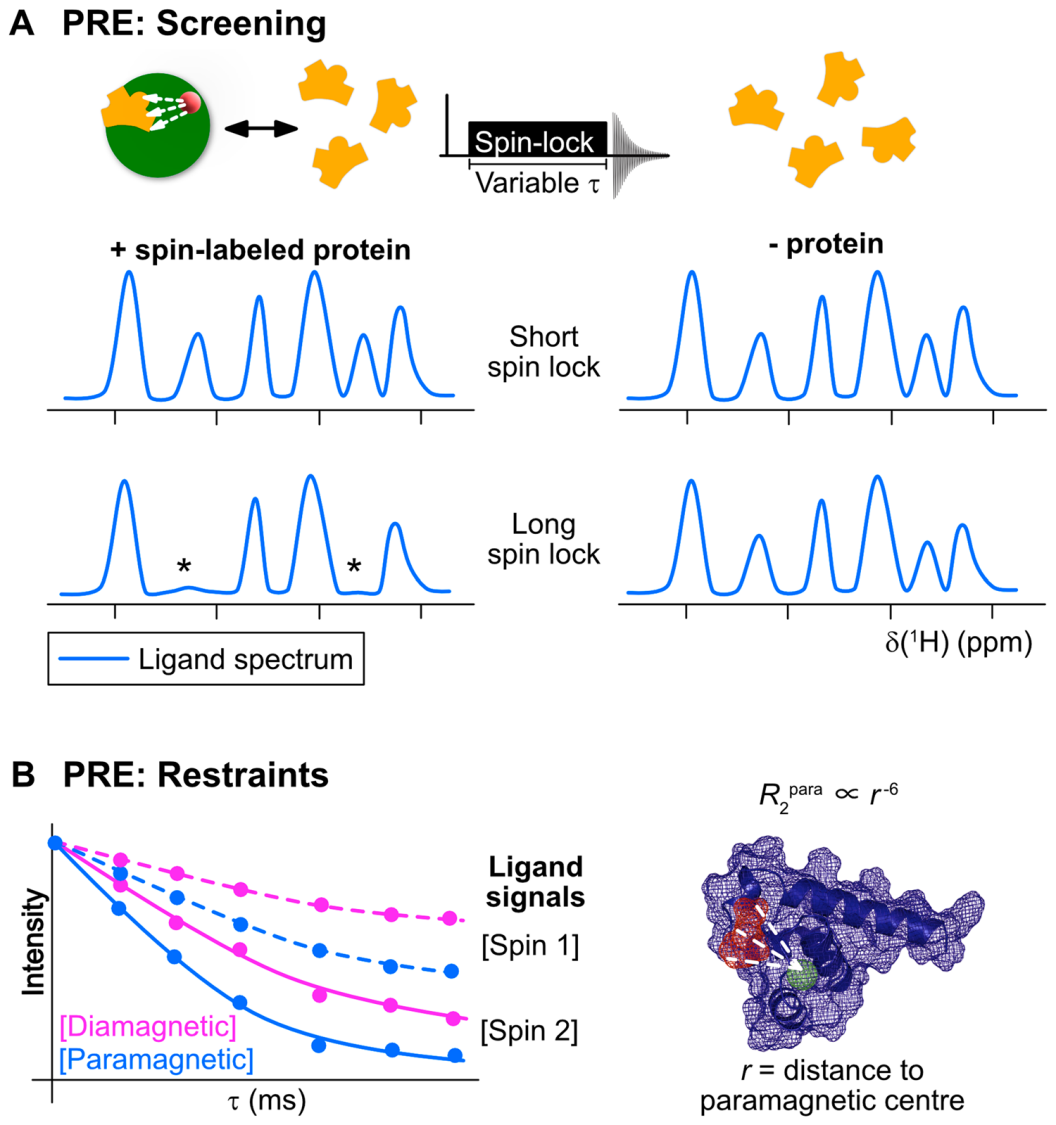
Applications of PRE in drug discovery. **a** Screening applications: Spectra for a mixture of ligands are compared with and without protein at short and long spin-lock durations ($\tau$), using a $T_{1\rho }$ spin-lock sequence. At long spin-lock durations, signals from ligands that interact with spin-labeled protein are broadened (*) due to the proximity to the paramagnetic center. This increases the observed $R_{2}$ rate by the amount $p_{{\text{b}}} \left( {R_{{2,{\text{bound}}}} + R_{{2,{\text{bound}}}}^{{{\text{para}}}} } \right)$ (Eq. 9). Non-interacting ligands do not experience significant attenuation. The spin-lock also serves to attenuate protein signals (not shown in the schematic figure) due to their faster transverse relaxation reducing overlap. **b** Structural restraints: Comparison of $R_{2}$ rates for the ligand signals in the presence of diamagnetic or paramagnetic-labeled protein may be used to determine $R_{2}^{{{\text{para}}}}$, which can be converted to a distance $r$ between the paramagnetic center (green mesh) and the ligand (red mesh) signal (Eqs. 1, 3)

For weakly binding ligands e.g. fragments, the experiment can be carried out in a ligand-observed fashion, which benefits from the narrow linewidths due to fast tumbling of the free ligand (rather than the slower tumbling of the protein complex). However, due to the strength of the PRE effect, even weakly-interacting molecules can be easily detected by line broadening of their signals upon addition of paramagnetic protein, compared to a diamagnetic reference, and furthermore, the protein requirements are very low (Fig. 6). These effects were demonstrated in two key papers in the early 2000s (Jahnke et al. 2000, 2001), and later for a protein with a native metal-binding site into which a paramagnetic ion could be exchanged (Bertini et al. 2004). In one approach a compound known to bind a target protein is modified to contain a paramagnetic label and is used to screen fragments binding at a second site, “second-site screening” (Jahnke et al. 2000), potentially allowing the two ‘hits’ to be linked to create a tighter binding compound. In this example only 10 μM Bcl-xL was required to detect a compound binding with a dissociation equilibrium constant *K*_d_ ≈ 1 mM at the second site. One-dimensional $R_{{1{\rho }}}$ measurements are used to detect the increased relaxation rate of interacting ligands, due to their reduced average distance to the spin label. This method is particularly robust against false positives: if the second ligand competes with the first, no signal will be seen as the two ligands must bind concurrently for a PRE effect to be observed. If there is no binding, no change in $R_{{1{\rho}}}$ rates between paramagnetic and diamagnetic samples will be observed since the average interaction distance with the spin-labeled ligand will remain much longer than if there is a binding interaction (Jahnke 2002). The method overcomes solubility problems typically associated with second-site screening, which can make it challenging to saturate a binding site with the known (first) ligand.

In a second paper, the use of PREs for primary screening was also demonstrated in a method known as SLAPSTIC (spin labels attached to protein side chains as a tool to identify interacting compounds) (Jahnke et al. 2001): the protein can be covalently tagged with a paramagnetic group, in this example using spin-labelling of lysines on FK506 binding protein, FKBP, and then used to screen a range of binding and non-binding compounds. Due to the dependence of the PRE on $r^{ - 6}$, spin-labeled residues need to be in the range ca. 10–20 Å from the binding site for ligand binding to benefit from the relaxation-enhancement effect without being broadened beyond detection, assuming the Solomon contribution is the dominant effect (Jahnke 2002). The observed relaxation rate is given by:

9 $$R_{{2,{\text{obs}}}} = \left( {1 - p_{{\text{b}}} } \right)R_{{2,{\text{free}}}} + p_{{\text{b}}} R_{{2,{\text{bound}}}} + p_{{\text{b}}} R_{{2,{\text{bound}}}}^{{{\text{para}}}} + R_{{2,{\text{ex}}}}$$

where $R_{{2,{\text{free}}}}$ and $R_{{2,{\text{bound}}}}$ are the diamagnetic relaxation rates of free and bound ligand, respectively; $R_{{2,{\text{ex}}}}$ is the exchange contribution due to exchange broadening from intermediate exchange; $R_{{2,{\text{bound}}}}^{{{\text{para}}}}$ is the paramagnetic relaxation of bound ligand and $p_{{\text{b}}}$ is the bound fraction. $R_{{2,{\text{ex}}}}$ can be typically ignored for weak binding ligands (high micromolar affinity) (Jahnke et al. 2001), since the paramagnetic contribution is calculated as $R_{{2,{\text{obs}}}}^{{{\text{para}}}} - R_{{2,{\text{obs}}}}^{{{\text{dia}}}}$, and the exchange contribution to the paramagnetic and diamagnetic $R_{2}$ rates is almost equivalent and so will be cancelled (Clore and Iwahara 2009). A term in $R_{{2,{\text{free}}}}^{{{\text{para}}}}$ can also typically be ignored. Considering calculations for the average distance between molecules in solution (a 1 M solution has an average center-to-center particle separation of 11.8 Å, and a 1 mM solution 118 Å) and considering estimates for the protein radius (10–30 Å for molecular weights 5–100 kDa) the average interaction distance of molecules at typical NMR concentrations (< 1 mM) is beyond the reach of both Solomon and Curie contributions (Erickson 2009). In addition, both contributions depend on the rotational correlation time, $\tau_{{\text{r}}}$, which for a small molecule in solution is on the order of 10 ps, further reducing the effect of these contributions. Consequently, this indicates that at typical NMR concentrations used for screening, $R_{{2,{\text{free}}}}^{{{\text{para}}}}$ is unlikely to be a significant contributor. At higher concentrations (approaching 1 M), $R_{{2,{\text{free}}}}^{{{\text{para}}}}$ could require consideration, however, this is unrealistic for biomolecular applications, and furthermore, the increased sensitivity of paramagnetic screening allows reduced sample concentrations. However, even weakly interacting molecules will see a substantial enhancement due to $R_{{2,{\text{bound}}}}^{{{\text{para}}}}$, which is determined by the tumbling time of the protein. This increases the sensitivity of paramagnetic screening to weakly binding fragments. Measurement of $R_{{1{\rho }}}$ rates, as described above, can allow differentiation between different ligands although the relaxation is affected by both distance from the PRE center and residence time.

In NMR experiments with protein–ligand complexes, interacting ligands experience an increased relaxation rate, $p_{{\text{b}}} R_{{2,{\text{bound}}}}$, due to the slower tumbling of the protein–ligand complex. In the case of SLAPSTIC, this effect is enhanced by the paramagnetic contribution $p_{{\text{b}}} R_{{2,{\text{bound}}}}^{{{\text{para}}}}$, benefitting from an approximately 50-fold relaxation enhancement at 12 Å as a result of the Solomon PRE effect (Jahnke 2002). This dramatically reduces the protein requirements with only 20 μM spin-labeled FKBP needed to detect interacting partners compared to 60 μM unlabeled FKBP. An $R_{{1{\rho }}}$ sequence was used to detect increased line-broadening of interacting ligands at longer spin-lock times, compared to non-interacting compounds, which do not experience the enhanced relaxation in the bound state. A particular advantage is that the PRE effect and $R_{{1{\rho }}}$ sequence quench protein signals, which otherwise can obscure ligand signals. Furthermore, $R_{{1{\rho }}}$ experiments can be acquired as a function of spinlock time giving a quantitative assessment of the relaxation rate for different ligands (Jahnke et al. 2000). Bertini et al. found that the use of a paramagnetic metalloprotein reduced the protein requirements for ligand screening by a factor of five to ten, depending on the binding affinity of the compound tested (Bertini et al. 2004). The effect on a CPMG experiment, where the increase in relaxation rate is dominated by the contribution from $R_{2}$ is increased by use of a paramagnetic metal ion in or near the binding site. With cobalt used in this case, the $R_{2}$ contribution is dominated by Curie relaxation. The $R_{2}$ enhancement under conserved conditions was shown to vary from a factor of 1.25 at 9 Å to 180 times at a 3 Å distance from the paramagnetic center. A similar screening application was demonstrated by using a two-point anchored lanthanide binding peptide on the SH2 domain of Grb2, bound to Gd^3+^, which like nitroxide spin labels, induces a PRE via the Solomon mechanism, although with S = 7/2, allowing a reduction in the protein requirements and increased sensitivity to weakly-binding ligands (Saio et al. 2011).

In addition to screening, PRE effects can also be used as a source of distance restraints to provide information on ligand pose in a binding site (Figs. 5, 6). To date, this approach has been less widely used than PCS restraints (discussed below) for protein–ligand applications. In the two examples described above, a possible source of distance information can be obtained from the differential relaxation of ligand protons, with protons closer to the paramagnetic center showing a faster $R_{2}$ rate. This requires knowledge of the position of the paramagnetic center as well as information on the $R_{2}$ rates in Eq. 9 in order to extract $R_{{2,{\text{bound}}}}^{{{\text{para}}}}$. Depending on the dominant relaxation mechanism, Eqs. 1 or 3 can then be used to extract distance information. When determining distances from transverse relaxation rates due to PREs from metals with a non-vanishing ${\Delta }\chi$, additional effects due to RDCs and DSA-CSA (dipolar shielding anisotropy-chemical shift anisotropy) cross-correlation effects can affect the PRE (Orton and Otting 2018). The latter can be minimized by measuring ^1^H spins with a lower CSA, compared to ^15^N for example, while the former can be reduced by measuring at lower magnetic fields and carefully selecting the lanthanide metal to reduce ${\Delta }\chi$ (Orton and Otting 2018). A careful choice of paramagnetic metal is important to accurately correlate PRE effects with distances. For example, transverse PRE for lanthanides with fast electronic relaxation times has minor contributions from the Solomon mechanism but is dominated by Curie relaxation (Orton and Otting 2018). Such challenges are mitigated for spin-labels with an isotropic $\chi$ tensor, such as nitroxide labels. In addition, intermolecular contributions may also affect the accuracy of distances determined from paramagnetic relaxation rates. This effect can be reduced by measuring samples at lower concentration (to minimize non-specific intermolecular interactions) within the sensitivity limits of the sample, although such effects can be hard to eliminate entirely (Orton and Otting 2018). Non-specific intermolecular effects are greatest for solvent-exposed regions. In fact the utility of non-specific intermolecular effects is demonstrated by solvent PREs to map surface interactions of complexes (Madl et al. 2009, 2011; Orton and Otting 2018). Solvent PREs using soluble PRE tags are used to map protein surfaces: here the soluble paramagnetic center is used at high concentration (mM) the paramagnetic center is typically Gd^3+^, which has a high spin state (S = 7/2), enhancing the Solomon PRE according to Eq. 1 and a slow electronic relaxation making the complex lifetime, described according to a second-shell interaction model with the complex assumed to tumble at the correlation time of the protein, significant for $\tau_{{\text{c}}}$ (Eq. 2). This results in significant PREs for surface exposed residues (Pintacuda and Otting 2002; Madl et al. 2009).

An example of PRE used to determine binding poses involves inhibitors of HIV-1 fusion, which bind a small hydrophobic pocket on the gp41 protein, which could not be crystallized with bound ligands. Using a spin-labeled peptide, which bound in an adjacent pocket, similar to the second-site screening approach (Jahnke et al. 2000), paramagnetic relaxation rates due to the Solomon mechanism were extracted by varying the concentrations of the receptor-peptide complex in the diamagnetic and paramagnetic forms in order to extract $R_{{2,{\text{bound}}}}^{{{\text{para}}}}$ (paramagnetic relaxation of the bound form) (Balogh et al. 2009). These data were used in combination with computationally docked ligand positions to select poses that match the PRE data. This was followed by an energy minimization calculation, using Xplor-NIH, incorporating the PRE data in the form of NOE distance restraints, leading to a consensus structure in agreement with the experimental data from one of the starting docked poses, and providing further information about the ligand–protein interactions (Gochin et al. 2011). The PRE-docked structures were also in good agreement with PCS data measured from the same tag with Co^2+^ chelated (Balogh et al. 2009).

## PCS in drug discovery

**Fig. 7:**
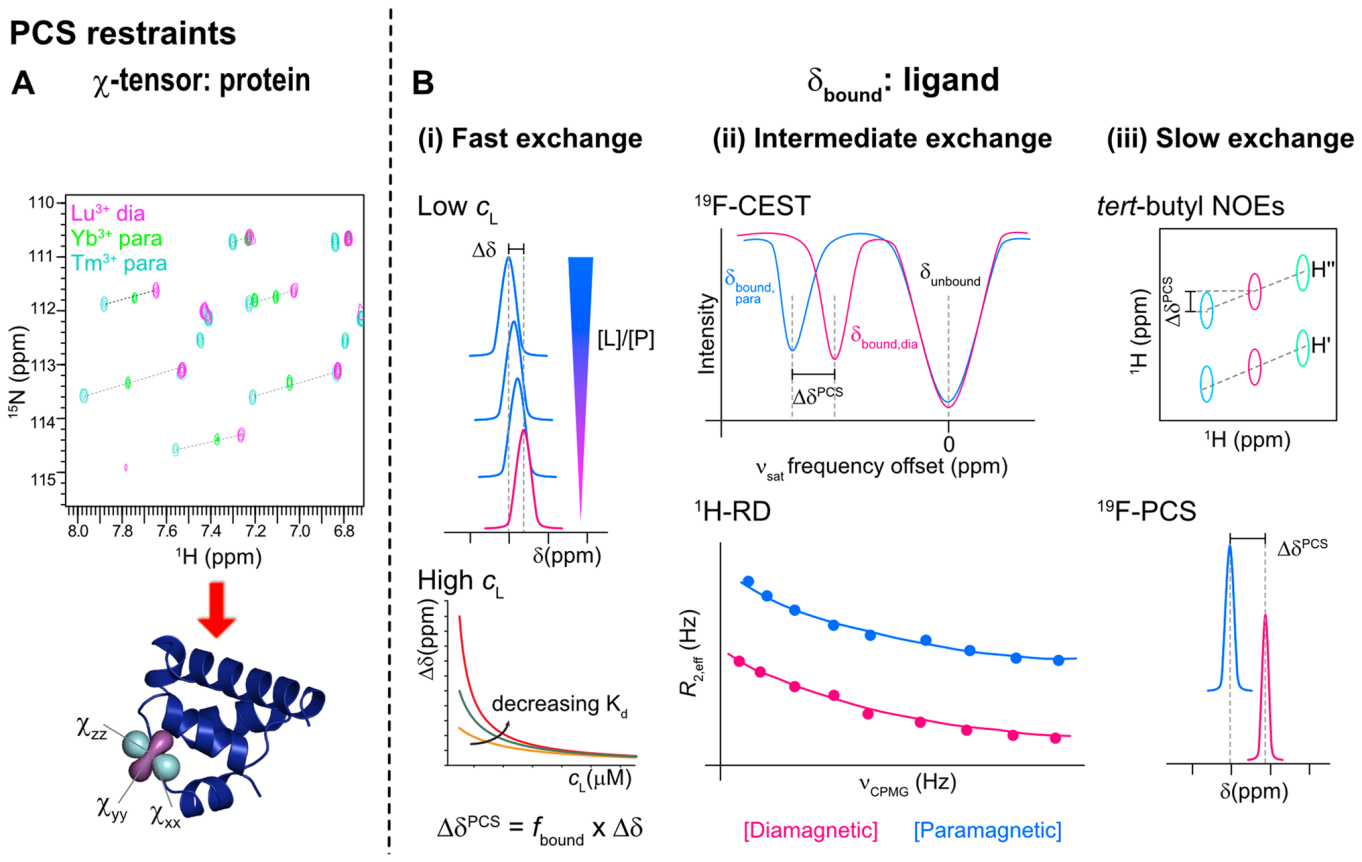
Applications of PCS in drug discovery. **a** Δχ-tensor: for spin- and isotope-labeled protein, the anisotropic magnetic susceptibility (Δχ-tensor) can be determined using standard 2D correlation experiments, e.g. ^1^H, ^15^N HSQC, using peak positions relative to the diamagnetic reference (e.g. Lu^3+^) to calculate the PCS shift (Δδ^PCS^), for each metal. Various software packages (Table 3) are available to calculate the paramagnetic tensor for each metal ion. **b** Determining or extrapolating the PCS for the (fully) bound ligand, δ_bound_: Restraints can be obtained by calculating the peak shift for ligand signals (Δδ^PCS^) relative to the diamagnetic reference, which is a result of the distance and orientation of a ligand residue with respect to the Δχ-tensor (Eq. 7). Different methods are required to determine δ_bound_ in the diamagnetic and paramagnetic cases in order to determine Δδ^PCS^ depending on the exchange regime of the ligand and saturation of the protein–ligand complex. (i) For ligands in fast exchange a titration may be used. Combined with knowledge of the equilibrium binding affinity constant, $K_{{\text{d}}}$, δ_bound_ can be extrapolated. At low ligand concentrations $\left( {c_{{\text{L}}} } \right)$, the ligand signal shifts towards δ_bound_ with increasing $c_{{\text{L}}}$. At high ligand concentrations, the ligand signal is dominated by the free ligand pool and Δδ^PCS^ decreases as a function of $K_{{\text{d}}}$ (Eqs. 10, 11). (ii) In intermediate exchange, the bound state signals are broadened beyond detection. For ^19^F ligands, ^19^F-CEST may be used: intensity reduction is observed relative to a reference spectrum as a function of the saturation frequency ($\upsilon_{{{\text{sat}}}}$). Intensity reduction is observed for the position of the free ligand (δ_unbound_) and the bound ligand (δ_bound_). For ^1^H signals, a relaxation dispersion experiment may be used and the profiles fit to determine the shift of the bound state. (iii) In slow exchange, the ligand is tightly bound and tumbles with the correlation time of the protein, leading to broad linewidths for the ligand signals. In ^1^H spectra, this renders the ligand signals indistinguishable from protein signals. Modifying the ligand to contain a tertiary butyl group (*tert*-butyl) leads to a strong, sharp signal, which is easily observed. 2D NOESY spectra allow identification of cross-peaks from the sharp *tert*-butyl signal to other ligand peaks, allowing determination of Δδ^PCS^, relative to the diamagnetic reference (magenta). If a ^19^F-labeled ligand is available, signals, whilst broadened, are easily detectable with no background signals, allowing easy determination of Δδ^PCS^

**Fig. 8:**
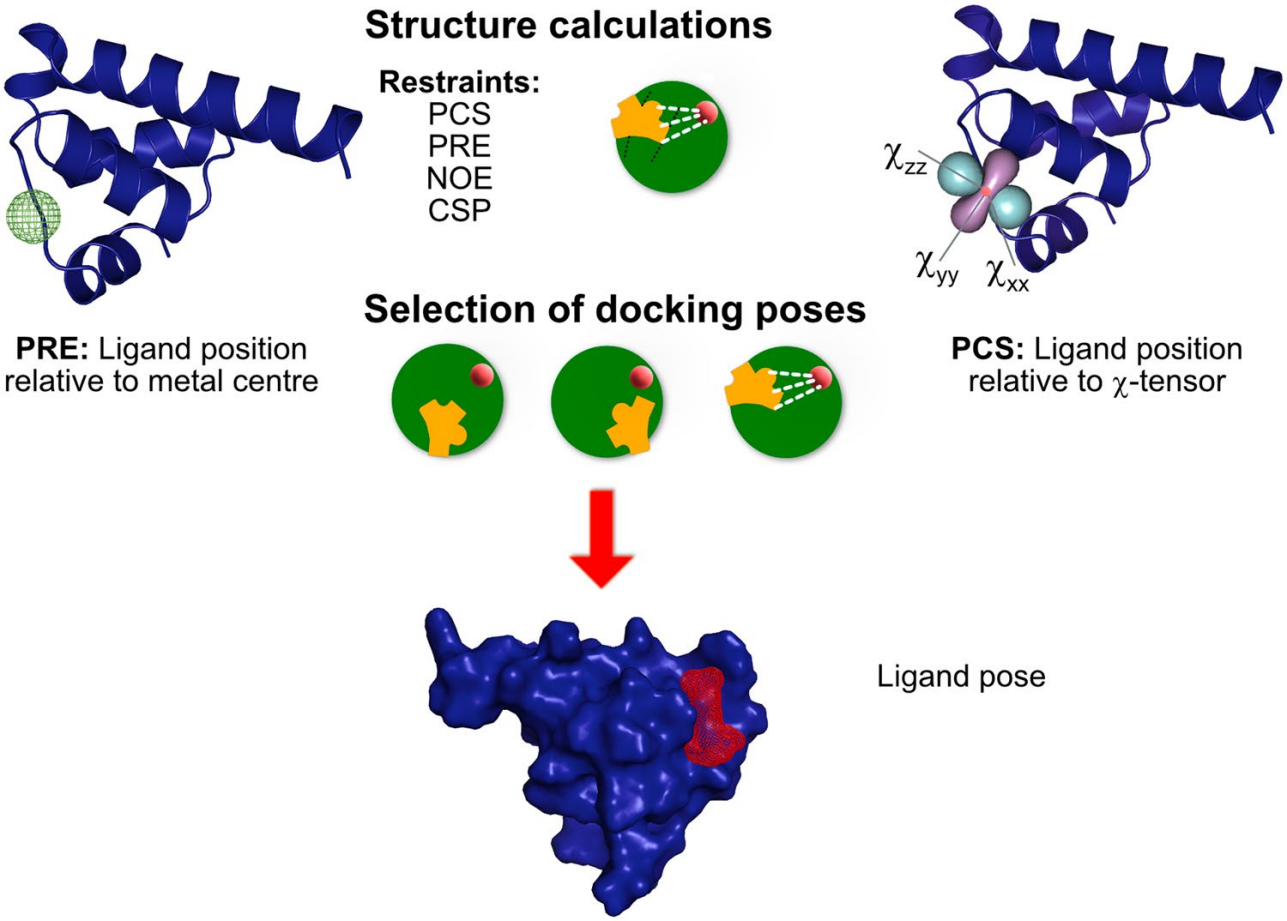
Calculation of ligand poses using paramagnetic data. PRE data including distance information (left) and PCS including distance and orientation information (right) may be used directly in structure calculations, potentially along with additional NMR restraints e.g. chemical shift perturbations (CSPs) and NOE restraints (if available) using molecular dynamics-based programs such as HADDOCK or Xplor-NIH (Tables 2, 3). Alternatively, docking software (Table 3) may be used to generate a selection of docking poses. Predicted Δδ^PCS^ values and/or distances to a paramagnetic center can be calculated for each pose, based on knowledge of the metal tensor or position of the paramagnetic center. The results are compared to the experimentally determined ligand shifts, allowing filtering or scoring of the computationally-generated poses and determination of the best fitting predicted ligand pose

PCS can be used in a variety of ways in drug discovery thanks to the provision of both distance- and orientation-dependent restraints, and the clear chemical shifts of the protein residues visible in 2D correlation spectra, without complex processing. Applications include compound screening and finding generalized binding site locations, as well as generating PCS-driven or PCS-filtered binding poses and structures (Figs. 7, 8). Different approaches to the tagging have been tried in these cases, with the tag applied to either the protein or to the ligand itself. As the effects are seen for all NMR-active nuclei, many have been explored, including ^1^H, ^15^N, ^13^C and ^19^F nuclei discussed below. As the PCS effect is dependent on $r^{ - 3}$, rather than $r^{ - 6}$ for PREs, structural information using PCS can be obtained at longer distances. PCS sufficient for use in calculations have been seen at distances as large as 38 Å for ^19^F ligands (Zimmermann et al. 2019), and it is expected that with newer tags, distances of 100 Å or even greater would also be possible (Joss and Häussinger 2019).

### Locating a binding site

A critical point on the drug discovery pathway is determining the position of fragment or ligand binding on the target protein. A variety of NMR-based methods can be used to determine the binding site (Gossert and Jahnke 2016; Sugiki et al. 2018; Nitsche and Otting 2018) but paramagnetism can also provide a highly sensitive approach.

Covalent attachment of a paramagnetic metal to a ligand or series of ligands induces PCS on simple protein-observed ^1^H, ^15^N or ^1^H, ^13^C correlation spectra (Otting 2010) upon binding to the protein. Provided that the diamagnetic protein spectrum assignments and a structure are available, the paramagnetic spectra can be assigned either manually or with software such as Echidna (Schmitz et al. 2006). The observed PCS can be used to calculate the effective tensor and the location of the paramagnetic center (Schmitz et al. 2008; Rinaldelli et al. 2015; Strickland et al. 2016; Orton et al. 2020).

The shifts can be used in a similar manner to chemical shift perturbations (CSP) in order to validate a hypothesized binding position. As the PCS are purely distance and orientation dependent and are not affected by (potentially allosteric) changes in chemical environment, they directly report on the true binding site, in contrast to CSP analysis, which can be affected by allosteric changes. An example of this approach is DOTA-tagged sevoflurane, which binds to calmodulin N- and C-lobes. The binding position was identified by calculating an effective ${\Delta }\chi$ tensor from the induced ${\Delta }\delta^{{{\text{PCS}}}}$ on the protein (Brath et al. 2015). The position of the calculated tensor, combined with knowledge of the maximum distance between the metal and the ligand, was used to map a sphere onto the protein, indicating the location of the ligand. The magnitude of the effective tensor was also used to qualitatively compare dissociation constants *K*_d_. In fast exchange, ligands with a lower *K*_d_ (higher affinity) have a greater population in the bound state leading to larger shifts on the protein, resulting in a larger effective tensor. A greater effective ${\Delta }\chi$ is seen in the C-lobe in comparison to the N-lobe of calmodulin, in agreement with previous affinity studies. Although in this case a similar *K*_d_ was observed by ITC for DOTA-tagged sevoflurane as for untagged sevoflurane, a concern is that the addition of such a large tag to a ligand could substantially alter its binding mode. It is also notable that in this case the *K*_d_ was not included in the calculations, which led to differences between the effective tensors in the two cases. The magnitude of these tensors are therefore not transferable to other systems with different *K*_d_ values, for example for screening applications. For such a purpose, the tensors must be calculated by either considering the *K*_d_ values or by calculating the tensor using the shifts seen on the signals of the tagged ligand. For the latter, as with all tensor calculations, a minimum of eight signals would be required.

While tagging the ligand enables detection of the binding region on the protein, tagging the protein allows PCS for the ligand signals to be detected, potentially allowing determination of the binding pose. The key challenge is then to determine the bound-state shifts for the ligand signals (δ_bound_) for use in structure calculations or filtering of docking poses. Different strategies have been employed in different exchange regimes, which are discussed below.

#### Weakly binding ligands, fast exchange

Tagging the protein of interest provides an opportunity to use transferred PCS to a ligand binding in fast exchange. Comparable to the PRE applications described earlier, the ligand experiences PCS in the bound state, with the effect observed as a weighted average of the time spent in the bound and free states (Fig. 7). Transferred PCS was demonstrated by the Otting group using a ligand in fast exchange binding a protein with a natural binding site for a metal cofactor (John et al. 2006). In the case that the total ligand concentration, $c_{{\text{L}}}$, is much greater than the concentration of protein, $c_{{\text{P}}}$, as in John et al. (2006), the fraction of bound ligand, $f^{{{\text{bound}}}}$, is:

10 $$f^{{{\text{bound}}}} = \frac{{c_{{\text{L}}}^{{{\text{bound}}}} }}{{c_{{\text{L}}} }} = \frac{{c_{{\text{P}}} }}{{K_{{\text{d}}} + c_{{\text{L}}} }}$$

with

11 $$\Delta \delta_{{{\text{PARA}}}}^{{{\text{obs}}}} = f^{{{\text{bound}}}} \Delta \delta_{{{\text{PARA}}}}^{{{\text{bound}}}}$$

12 $$\Delta v_{{{\text{PARA}}}}^{{{\text{obs}}}} = f^{{{\text{bound}}}} \Delta v_{{{\text{PARA}}}}^{{{\text{bound}}}}$$

$\Delta \delta_{{{\text{PARA}}}}^{{{\text{bound}}}}$ and $\Delta v_{{{\text{PARA}}}}^{{{\text{bound}}}}$ in Eqs. 11 and 12 represent the PCS and paramagnetic relaxation contribution, respectively, that occur in the fully bound state. For lanthanides, $\Delta v_{{{\text{PARA}}}}^{{{\text{bound}}}}$ is dominated by Curie relaxation (Eq. 5) (John et al. 2006).

This assumes short electron relaxation times (as found for lanthanides) and long $\tau_{r}$ (rotational correlation time) at high magnetic fields. $\Delta \delta_{{{\text{PARA}}}}^{{{\text{obs}}}}$ and $\Delta v_{{{\text{PARA}}}}^{{{\text{obs}}}}$ represent the observed shift and line broadening, respectively. From Eqs. 10–12, plotting $\Delta \delta_{{{\text{PARA}}}}$ or $\Delta v_{{{\text{PARA}}}}$ against $\frac{{c_{{\text{L}}} }}{{c_{{\text{P}}} }}$ allows calculation of the $K_{{\text{d}}}$ and $\Delta \delta_{{{\text{PARA}}}}^{{{\text{bound}}}}$ or $\Delta v_{{{\text{PARA}}}}^{{{\text{bound}}}}$ respectively from 1D ligand titration spectra. The PCS, along with PRE if measured, of the bound state can then be input into one of several software packages that integrate PCS data processing into docking or scoring in order to determine the binding position (Table 3). The addition of line-broadening information to the PCS data, due to the dependence on $r^{ - 6}$, is particularly beneficial for short-range interactions, where the resolution of PCS data is lower; it is of most use on spins with small γ, commonly ^15^N and ^13^C, as the peaks are not attenuated as drastically compared to ^1^H nuclei, although care must be taken due to the effect of chemical shift anisotropy on heteronuclear PRE, that can even lead to negative PRE effects (Orton et al. 2016). This can however be easily calculated and available software accounts for this in calculation of tensors and predicted PRE values for a given structure (Orton et al. 2020).

**Table 3:** Software used in paramagnetic-NMR applications discussed in this paper

Software	References	Description	Paramagnetic restraints
Xplor-NIH	Schwieters et al. (2003)	Structure determination program for use with NMR, X-ray and neutron scattering data	
PARArestraints	Banci et al. (2004)	Module for adding paramagnetic restraints into Xplor-NIH	PCS, RDC, PRE, cross-correlated relaxation rate (CCR)
AutoDock Vina	Trott and Olson (2010)	Small molecule docking	
AutoDock 4.2	Morris et al. (2009)	Small molecule docking, previous generation to AutoDock Vina	
HADDOCK	Schmitz and Bonvin (2011) and Van Zundert et al. (2016)	Docking, including possibility to add paramagnetic, CSP and other restraints e.g. distance restraints (ambiguous or unambiguous) to guide docking	PCS, RDC
DOCK6 UCSF	Allen et al. (2015)	Small molecule docking	
Rosetta	Schmitz et al. (2012) and Kuenze et al. (2019)	Structure calculation of proteins, protein–ligand and protein–protein complexes, including paramagnetic restraints	PCS, RDCs, PREs
Echidna	Schmitz et al. (2006)	Assignment of paramagnetic ^15^N HSQC using assigned diamagnetic spectrum and crystal structure	PCS
Paramagnetic CYANA	Güntert (2004) and Barbieri et al. (2004)	Structure determination, including use of paramagnetic data as structural restraints	PCS, RDC, CCR
Numbat	Schmitz et al. (2008)	Fitting of magnetic anisotropy tensor from PCS values and a PDB stucture	PCS
Paramagpy	Orton et al. (2020)	Fitting and visualisation of tensors based on experimental data	PCS, RDC, PRE, CCR
FANTEN	Rinaldelli et al. (2015)	Web-based calculation of magnetic anisotropy tensor from PCS and/or RDC data. Multiple metals can be simultaneously fitted	PCS, RDC

Calculations using these data, as with all calculations involving a single anisotropic magnetic susceptibility tensor, lead to up to fourfold degeneracy in the position of the calculated ligand due to the symmetry of the tensor itself. In this example, both ^1^H and ^13^C spectra of the ligand were measured in order to maximize the available restraints (John et al. 2006). The calculated ligand position was in good agreement with the binding position of a structurally similar ligand seen in crystal structures, demonstrating the validity of the method for this case.

Other examples have been demonstrated using tagged proteins. A lanthanide binding peptide tag covalently linked to a native cysteine via an intramolecular disulfide bond on the SH2 domain of Grb2 (Saio et al. 2009) was used to measure $\Delta \delta^{{{\text{PCS}}}}$ to both a high affinity ligand (a macrocyclic inhibitor) and low affinity peptide (pYTN), in combination with Xplor-NIH PARA-restraints module (Schwieters et al. 2003; Banci et al. 2004). Knowledge of the $K_{{\text{d}}}$ from titrations was used to extract $\Delta \delta^{{{\text{PCS}}}}$ for the ligand in fast exchange, while for the ligand in slow exchange, deuterated protein was used to observe the bound-state shifts (Saio et al. 2011). Although only one tag position was used, four metal ions provided different tensors. A similar approach with ligands in fast exchange was demonstrated on FKBP-12, using a two-point anchored CLaNP-5 tag. Three tag positions were used and $K_{{\text{d}}}$ from NMR titrations enabled estimation of $\Delta \delta^{{{\text{PCS}}}}$ using Xplor-NIH PARA-restraints module, resulting in an average RMSD to the NOE-calculated ligand position of 2.8 ± 0.4 Å (Guan et al. 2013).

#### Tight binders, slow exchange

In the case of slow exchange, typically seen for high-affinity inhibitors in advanced drug discovery programs, separate NMR signals are observed for the bound and unbound ligand in the ratio seen in solution. This is advantageous for the use of PCS as the shift induced (i.e. $\Delta \delta^{{{\text{PCS}}}}$ in Eq. 7) can be directly read out from the spectrum and used in further calculations (Saio et al. 2011). If the exchange is on a suitable timescale, exchange spectroscopy (EXSY) can be used to assist with assignment (Jeener et al. 1979; John and Otting 2007). However, the challenge in such cases is that the ligand tumbles with the overall correlation time of the protein and so no benefit is obtained from the faster tumbling of the free ligand pool, i.e. the transferred PCS effect is lost, leading to broader linewidths.

Chen et al. present an approach using a ligand decorated with a *tert*-butyl group, which benefits from rapid bond rotation and nine equivalent protons, leading to an extremely narrow linewidth (Chen et al. 2016). The *tert-*butyl signal can be observed in NOESY spectra, allowing ${\Delta }\delta^{{{\text{PCS}}}}$ to be calculated, and may enable identification of other ligand peaks due to NOE transfers (Fig. 7). Together the ${\Delta }\delta^{{{\text{PCS}}}}$ data can be used to position the *tert*-butyl group and other parts of the ligand where NOEs are identified. In this example ligand PCS was used in combination with docking poses to select a confirmation interacting with dengue virus protease. In cases with tight binding compounds where crystallography proves intractable, this approach may be invaluable.

A ^19^F-PCS approach has recently been demonstrated by the Häussinger group using tightly-binding ^19^F-labeled sulfonamide inhibitors of human carbonic anhydrase as models for a tight-binding system, with attachment of the rigid DOTA-M8-SSPy and M7PyThiazole-DOTA tags at five different sites with Ser to Cys mutations (Zimmermann et al. 2019). Due to the lack of background in ^19^F spectra, simple 1D ^19^F spectra can be used to detect ${\Delta }\delta^{{{\text{PCS}}}}$, with lanthanide-fluorine distances up to 38 Å detectable, leading to unambiguous localization of the inhibitors (Fig. 7). The distance range is considerably longer than that achieved in ^1^H NMR (typically up to 25 Å). Accuracy of the ^19^F placement approaches 0.8 Å in some cases, compared to X-ray structures (Kim et al. 2000). However, the authors demonstrate that the accuracy depends on the number and choice of tensors, and the angle between them.

#### Intermediate exchange

Many lead or lead-like compounds do not bind tightly enough to enter the slow exchange regime, but also do not benefit from fast exchange observed with fragments. This leads to the challenging prospect of structure determination in intermediate exchange, with severe line broadening in this regime limiting the ability to track ligand shifts easily for $K_{{\text{d}}}$ determination, preventing determination of the bound-state shifts using methods described above. Despite this, several approaches have been taken to determine the bound-state shift, and hence ${\Delta }\delta^{{{\text{PCS}}}}$, indirectly.

One approach involves using chemical exchange saturation transfer (CEST) (Vallurupalli et al. 2012) to identify the population of bound ligand where the bound signal is broadened beyond detection (PCS-CEST) (Fig. 7) (Gao et al. 2017). ^19^F mono- and di-fluorinated inhibitors of the BRM bromodomain were used due to interference by ^1^H-^1^H NOEs on ^1^H CEST measurements (Bouvignies and Kay 2012). By using a large molar excess of the ligand over the paramagnetically tagged protein (1:0.025), the ^19^F signal was only slightly reduced in intensity, despite the severe line broadening effect. Such a sample could then be measured by ^19^F CEST. In brief, this involves a swept saturation frequency ($\upsilon_{{{\text{sat}}}}$) in the ^19^F dimension, with dips in intensity compared to a reference spectrum highlighting the shifts of both the high-populated unbound state and the low-populated bound state at given saturation frequencies (Fig. 7). Shifts of up to 2 ppm were detected in this way; these were then compared with back-calculated PCS of the bound-state ligand from ligand poses that were calculated using HADDOCK (Schmitz and Bonvin 2011). Alternatively Xplor-NIH (Schwieters et al. 2003) with PARA-restraints (Banci et al. 2004) may be used. Whilst two of the clusters were indistinguishable from one another when only one fluorine atom was present, validation with a difluorine analogue was able to determine the best cluster. This method is also viable in a situation with a ligand that is not in intermediate exchange but has low solubility as the bound state can be determined, even when it is very weakly populated.

If ligand ^19^F atoms are unavailable, relaxation dispersion has been demonstrated as an alternative method to find the bound PCS of compounds in intermediate exchange—PCS-RD (Fig. 7) (Xu et al. 2018a). By implementing the “perfect echo” element (Aguilar et al. 2012) in a relaxation dispersion (CPMG) experiment to prevent the evolution of homonuclear scalar couplings, the ^1^H PCS of the sparsely populated state could be determined. In the same manner as above, this allowed filtering of docked poses (in this case by AutoDock (Morris et al. 2009; Trott and Olson 2010)) by the quality of the fit of back-calculated PCS values to the observed experimental values. In this case, only four PCS values were used, leading to four possible clusters after analysis, potentially due to too few data points.

### Degeneracy of the tensor and solutions

A key challenge when using PCS restraints is the degeneracy of the magnetic susceptibility anisotropy tensor, which leads to multiple solutions, as well as the intrinsic triangulation problem (Fig. 5) (Bertini et al. 2016). In some cases, structural knowledge of the system can eliminate the degeneracy, for example in the case of steric clashes, locations away from the protein surface or chemically unfeasible interactions. Saio et al. determined their final structure possibilities by first determining the best 20 structures based on ‘PCS energy’, before reducing these to ten using more traditional binding energy calculations, thereby removing implausible results that could come as a result of degenerate positions (Saio et al. 2011).

However, to directly calculate one single position, rather than eliminating artificially the false results, multiple data sets with different tensors must be used, either using multiple tag positions (Zimmermann et al. 2019) or tags with orthogonal tensors, for example chiral compounds (Lee et al. 2016) or otherwise sufficiently different chelation. Zimmerman et al. showed that the degree of orthogonality of the tensors affected the accuracy of the positioning of the atom, in their case using ^19^F-PCS data. Using four tensors gave very good agreement with crystallographic structure determination. An angle score was calculated using the intersection of the normal vectors to the tensor isosurfaces (surfaces along which the observed shift is equal for any point) at the intersection point. This gave a readout of orthogonality that could then be used to directly compare data calculated with a variety of sets of three tensors. The authors found that for three iso-surfaces with an angle score below 30°, the calculated position closely matches the four-tensor calculation, but with an angle score above 40°, this could lead to a deviation of up to 10 Å.

### PCS reliability and effect of tag mobility

When using paramagnetic methods, there are limitations that should be considered. The effect of tag mobility on the tensor and PCS back-calculations has been modeled (Shishmarev and Otting 2013) using a spherical protein model attached by a single tether with various motional relationships to the paramagnetic center. It was shown that a back-calculated effective tensor describes the shifts seen on protein atoms well, even with the model representing the largest tag flexibility. The authors also showed that while the PCS predictions for atoms within the protein sphere were consistent with calculated values, predictions for atoms above the surface of the protein model (i.e. outside of the modeled sphere) were worse, indicating that for protein–protein and inter-domain structure determination, the predictions are less accurate. This could have consequences, for example, when studying peptide-protein interactions or for larger ligands that do not sit deep in binding pockets, but should not affect ligands seen in deep pockets, which therefore would lie within a spherical protein model (Shishmarev and Otting 2013). The amplitude of motion of the tag and the length of the tether were found to be key parameters. The amplitude of motion is, however, difficult to quantify with a simple experimental setup as the authors show that comparison of the calculated tensors fitted with RDC and PCS values is not a good measure.

### Tag tensor prediction

Determination of the position of the paramagnetic center is important for the precision and accuracy of paramagnetic restraints. For two-armed tags, the position of the metal is much more restrained than that in single armed tags and the mobility of the tensor is highly restricted. Where no isomerization of the tag can occur, this can lead to a tensor magnitude and position that, with a given metal, is highly consistent between the free tag and when it is applied to different proteins (Keizers et al. 2008; Xu et al. 2009; Lee et al. 2017). These can then be used directly in calculations without back-calculating the tensor or its position, with the advantage that full protein assignments are not required. Guan et al. showed that with CLaNP-5, the ligand position calculated with the predicted tensor was in good agreement with the position calculated with NOEs (Guan et al. 2013). Whilst the calculation using the predicted tensor gave a slightly worse fit to the NOE-calculated result than that calculated with a newly defined tensor, the data were sufficient for analysis of possible binding modes and determination of the binding site of the ligand once unrealistic degenerate models were removed. However it should be considered that not all double-arm lanthanide tags are rigid and some also undergo isomerization, which would prohibit such a method from being used in these cases (Hass et al. 2010). The binding location of the tag can also cause further flexibility and increase the error in the calculations.

## Conclusions and perspectives

In recent years various applications of paramagnetic NMR in drug discovery have been reported. PRE-based screening allows more sensitive detection of weak binding with reduced protein concentration, while PCS restraints have been used in multiple applications to allow localization of ligands in different exchange regimes. This appears especially useful in early stages of drug discovery involving weakly binding fragments, which are often difficult to crystallize. Paramagnetic restraints can be used for structure calculations of protein–ligand complexes, using e.g. Xplor-NIH or HADDOCK, or to filter docking poses derived from computational analyses (Fig. 8). Given the challenge of obtaining sufficient restraints for accurate structure calculations, filtering of docking poses is often preferred and more time-efficient. Applications of ligand placement have primarily used PCS restraints due to the ease of tensor determination from 2D correlation spectra of the protein target. However, it can be challenging to extract $\Delta \delta^{{{\text{PCS}}}}$ if accurate information on the binding affinity (*K*_d_) is unavailable, or if the binding kinetics is in intermediate to slow exchange where ligand signals are challenging to detect. Methods to overcome these difficulties have been proposed, but likely remain beyond the scope of many projects. PRE restraints are less powerful for structural analysis of protein–ligand complexes since they only provide distance and no orientation restraints, and the accuracy of the distance information depends on the ability to extract $R_{{2, {\text{bound}}}}^{{{\text{para}}}}$ as well as tag flexibility. Nevertheless, in applications where rapid filtering of possible ligand binding poses is required, PREs may provide a time-efficient approach to score and enhance further development of ligands. In conclusion, paramagnetic restraints can complement standard methods of structure-based drug design, with opportunities for more sensitive screening, filtering of computational docking poses, and where required, calculation of ligand–protein structures. Further developments of paramagnetic approaches will conceivably enhance NMR-based drug discovery.
